# Why food insecurity persists in sub-Saharan Africa: A review of existing evidence

**DOI:** 10.1007/s12571-022-01256-1

**Published:** 2022-02-03

**Authors:** Vibeke Bjornlund, Henning Bjornlund, André van Rooyen

**Affiliations:** 1 UniSA Business School, University of South Australia, Adelaide, Australia; 2 International Crops Research Institute for the Semi-Arid Tropics, Addis Ababa, Ethiopia

**Keywords:** Food security, Food crops, Export crops, Food production systems, Hunger and malnutrition, Rural development

## Abstract

This article is the third in a series of historical reviews on sub-Saharan Africa (SSA), exploring why agricultural production and irrigation schemes are underperforming, and how this contributes to high levels of food insecurity. The expression ‘food security’ emerged in 1974 following the Sahel and Darfur famines. Despite SSA being a net agricultural exporter, food insecurity has persisted and is increasing. This is largely a legacy of the export-oriented colonial agricultural production systems, which procured scarce fertile land, water and labour to meet the needs of industries and consumers in the Global North. Colonialism also undermined the social contract between traditional leaders and communities, which had been instrumental in managing food scarcity in earlier times. Post-independence, agricultural policies remained focused on exports and neglected critical research and investment: integrating food productions systems into the domestic economy; developing supply chains and associated market, storage and value-adding infrastructure; and introducing appropriate technologies. As a result, Africa is the only region in the world where increased export production caused a decline in per capita food production. African nations should be extracted from the debt accrued due to poor colonial investments, World Bank lending practices, and global currency and interest fluctuations, which have crippled their capacity to support agriculture and improve livelihoods and food security. Farming needs to be profitable, which includes farmers being connected to domestic supply chains and market signals, local value-adding, and post-harvest storage. This will create jobs and increase income earning capacity, which is the key to households’ food security.

## Introduction

Food security is a critical part of the Sustainable Development Goals and government policies in sub-Saharan Africa (SSA). The expression “*food security*” was introduced in 1974 following famines in the Sahel and Darfur (Gerlach, 2015), and was initially focused on the production and availability of sufficient food. However, these and later African famines were not due to unavailability or insufficient regional production of food. Rather, they arose from governments’ failure to integrate and keep track of rural food production, storage and distribution systems, and governments inability or unwillingness to provide grain in a timely manner (de Waal, 2018; Lappé & Collins, 1982; Sen, 1981). Food security is, therefore, inextricably linked to poverty, governments’ capacity, and political willingness to mediate (Reutlinger & Selowsky, 1976).

The concept of food security now encompasses physical and economic access to sufficient, safe, and nutritious food, which meets dietary needs and food preferences for an active and healthy life (FAO, 1996). However, many households in SSA have been unable to produce or buy sufficient nutritious food. Access to food across SSA improved in the 1960s and 70 s with the availability of US grain imports. However, the debt crisis and International Monetary Fund’s (IMF) Structural Adjustment Policies (SAPs), imposed in the 1980s and 90 s, curtailed spending on imports, and the availability of food fell below levels in the 1960s (Dietz, 2013). The situation has slowly improved since 2000, but in 2016, 27% of the population lived with severe food insecurity due to rises in inequality and income disparity (Dietz, 2013; FAO, 2017). This is affecting more than 330 million people with 240 million being malnourished, particularly rural households (FAO, 2017). A five-fold increase in population since independence has exacerbated the problem, leaving SSA four times more affected than any other region and with food insecurity increasing (FAO, 2017; van Bavel, 2013). This situation has arisen despite most SSA countries being net agricultural exporters. The focus on large farms and western technology in agricultural policies for national food sovereignty, has meant that rural economic development has been neglected (Fox & Jayne, 2020). Recognising the influence of global political and market forces on food prices and availability, Watts and Bohle (1993) recommended four national food policy goals that remain valid: i) producing or importing enough food; ii) monitoring regional availability of, and securely storing and distributing, food during shortages; iii) shielding people from erratic and distorted market fluctuations and interference by special interest groups; and iv) creating food production and income earning opportunities for its people.

Reports about food insecurity in SSA commonly discuss the complex and interrelated contributory factors, such as low production and yields, primitive tools, lack of credit and investment in infrastructure, government policies, and corruption. This neglects how the colonial system instituted economic dependency, which was further entrenched post-WWII through global economic integration. For more than 60 years, international funding agencies have focused on agriculture as a predominantly technical issue and attempted to address the contributory factors without considering the historical context; hence, solutions have remained elusive.

In two previous papers we substantiated the argument that SSA was food secure prior to colonialism (Bjornlund et al., 2020a, b). When scarcity occurred, it was transitory and caused by drought, flooding, pests, or conflicts, rather than maldistribution of resources (Parsons & Palmer, 1978). Eyewitness accounts from the 1400 s through to slave records of the 1800s suggest that Africans were well-nourished, strong and of regular stature (Diffie et al., 1977; Hawthorne, 2003). Food security was enabled by endogenous agricultural, social, and trading systems, prioritizing diversity, adaptability, mobility, and connecting different ecological zones (Amin, 1973). These systems managed the limitations and challenges imposed by SSA’s biophysical environment (Bjornlund et al., 2020a, b).

In this paper we provide two arguments for the persistence of food insecurity in SSA. First, colonialism fundamentally disrupted and suppressed the existing food security systems, resulting in widespread poverty, chronic food shortages and malnourishment. By focussing political and economic development on resource extraction and neglecting the biophysical limitations, environmental degradation and social inequality increased, and unpredictable rainfall events now led to human catastrophes (Morgan & Solarz, 1994). Second, the post-independence governments continued the expansion of non-value-added exports to the Global North. Investment in African institutions and policies, to foster growth and equitable employment in domestic and regional economies, was neglected. International institutions were dominant and served the political and economic interests of the Global North. We substantiate our two arguments in three sections by describing the: i) exogenously driven colonial systems’ impact on food security; ii) transition to independence without the required social transformation to secure political and economic accountability from the new governments; and iii) post-independence developments, which further increased export crop production and failed to support domestic food production and small-scale farmers.

As with most papers dealing with Africa from pre-colonial times to the present, this paper depends on the limited data collected by colonial administrations and African governments (Manning, 2010). As Africa’s resource endowments and biophysical and socioeconomic conditions were diverse, and colonial structures differed in patterns and organisations, the impact of colonialism varies spatially. This paper focuses on the general trends in external political and economic impacts and local policies, rather than the nuances of development across the region. Wherever possible, we use present day names for countries and geographic areas.

## Colonialism: capturing resources

European nations divided Africa between them to prevent conflict from the increasing competition for resources. The Berlin Conference in 1884 gave each European nation exclusive control over separate territories (Michalopoulos & Papaioannou, 2017). Custom duties were the preferred revenue, and infrastructure investments were restricted to ports, railroads, and roads to facilitate exports (Hopkins, 1973; Jedwab & Moradi, 2016). Section 2.1 outlines the colonial administration’s role to facilitate exports by securing land and labour, enforcing law and order and raising revenue through indirect rule, and the three implementation models used by the colonial powers. Section 2.2 covers the impact on food security.

### Colonial administration system

#### Securing land and labour

Securing land and labour for export crop production was hampered by existing food production systems, cottage industries and domestic trade (Austin, 2008; Binswanger et al., 1995). European access to land, mines, and forests was achieved by vesting land and resources in the administrations (Potts, 2012). African farmers’ land access became conditional on the delivery of export crops, and food requisitions for the mines, public works, and colonial administration (Guyer, 1978). To coerce Africans to engage in wage labour, local processing and cottage industries were discontinued (Frankema & Waijenburg, 2012) and colonial taxes could only be paid in new colonial currencies (Ochonu, 2006; Saul, 2004). Tax measures were used (de Haas, 2017); for example, hut taxes could only be paid in cotton in Sierra Leone, and taxes were lower if paid in cotton in Mali (Robins, 2016). These policies transferred fertile land and labour to the production of export crops, regardless of households’ food needs.

#### The indirect rule system

The European administrations involved very few people (Kirk-Green, 1980), which limited their ability to enforce new institutions. Hence, France and Britain used “indirect rule” by adapting existing institutions to suit their needs. Native authorities were given the responsibility for tax collection, export quotas, food requisitions, and labour recruitments (Boone, 2003; Mamdani, 1996). Chiefs were incentivized by receiving a percentage of the taxes (Frankema et al., 2014), and the threat of losing their privileges if they failed to comply. The economic rewards and the ability to grant privileges gave chiefs powers above their traditional roles, creating a wealthy elite that was loyal to the colonial administration (Heldring & Robinson, 2012; Tignor, 1993). This shift in the role of native authorities undermined the social fabric of trust and inter-dependency.

The pre-colonial labour systems of debt bondage and slavery were integrated into the administrative framework and facilitated the rapid development of the cash-crop economy (Berry, 1993; Frankema et al., 2014). While the colonial metropoles could not endorse slavery, abandoning it would have exacerbated labour shortages; hence, slavery remained an integral part of the colonial production system (Freund, 1984).

#### Three implementation models

**Fig. 1:**
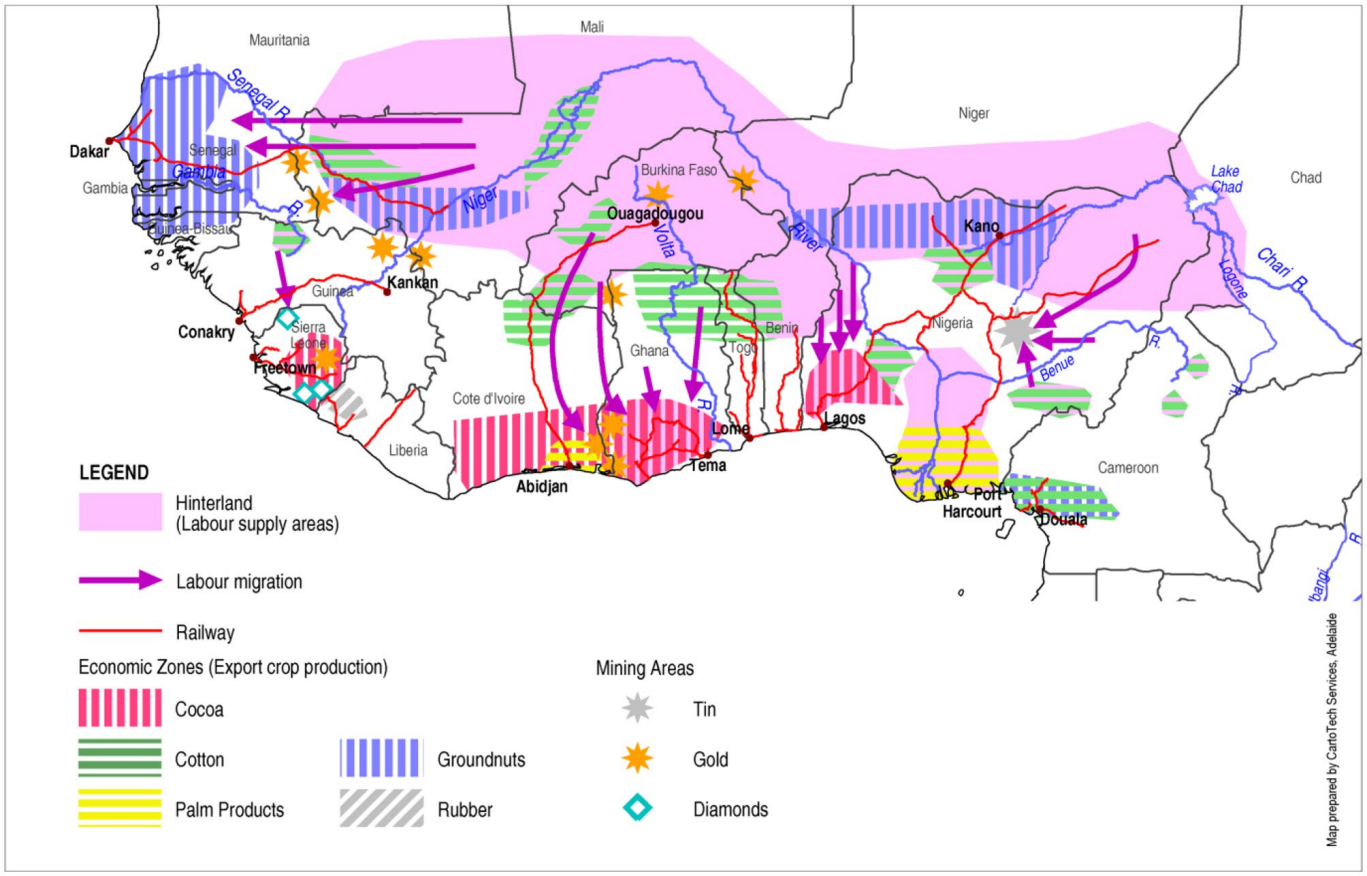
Export crop production and labour migration in Western Africa 1920s. Adapted from Austin (2008)

**Fig. 2:**
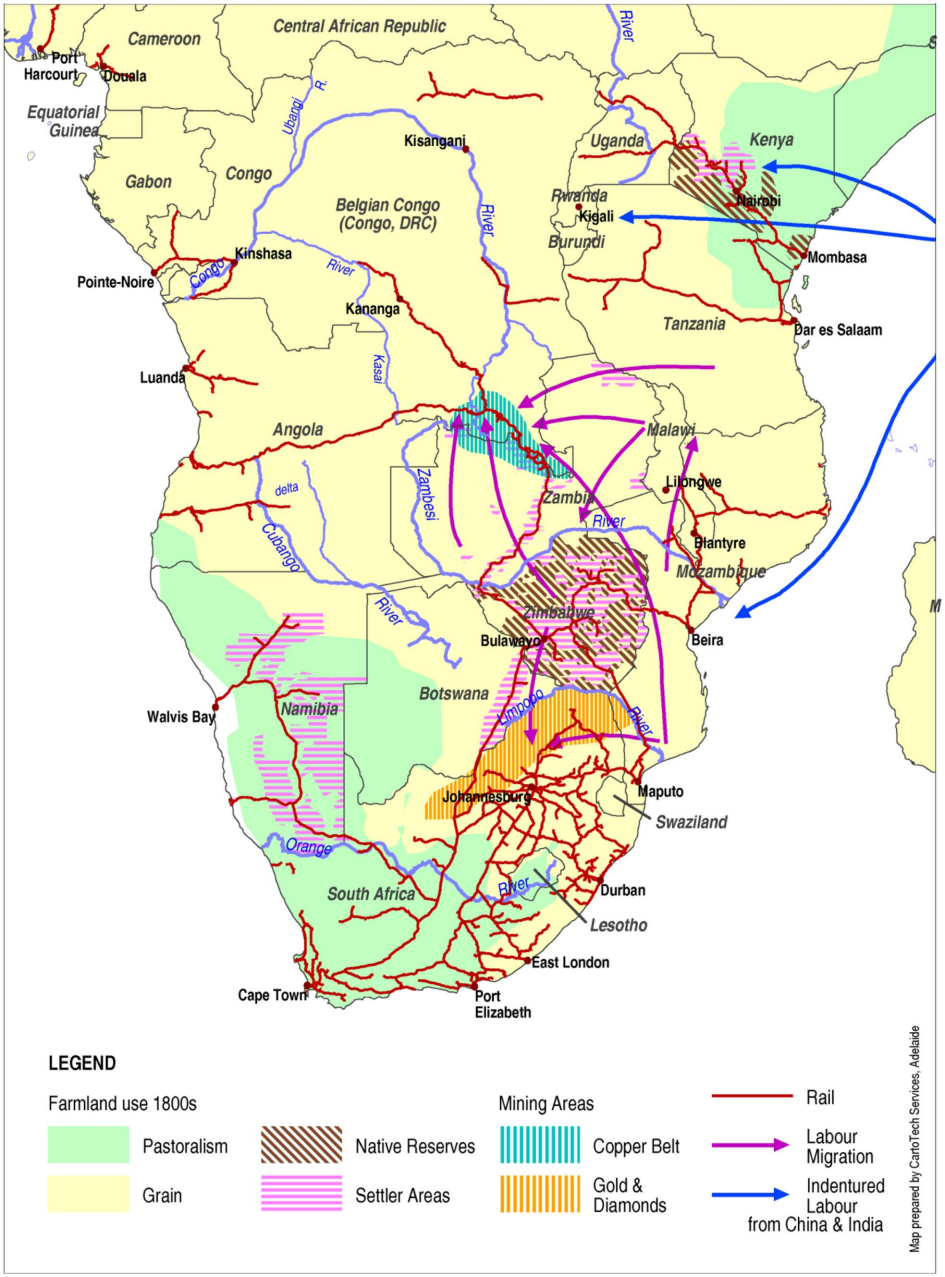
Settler and mining areas, labour pools, and migration, 1950s ( Source: map compiled by authors)

**Fig. 3:**
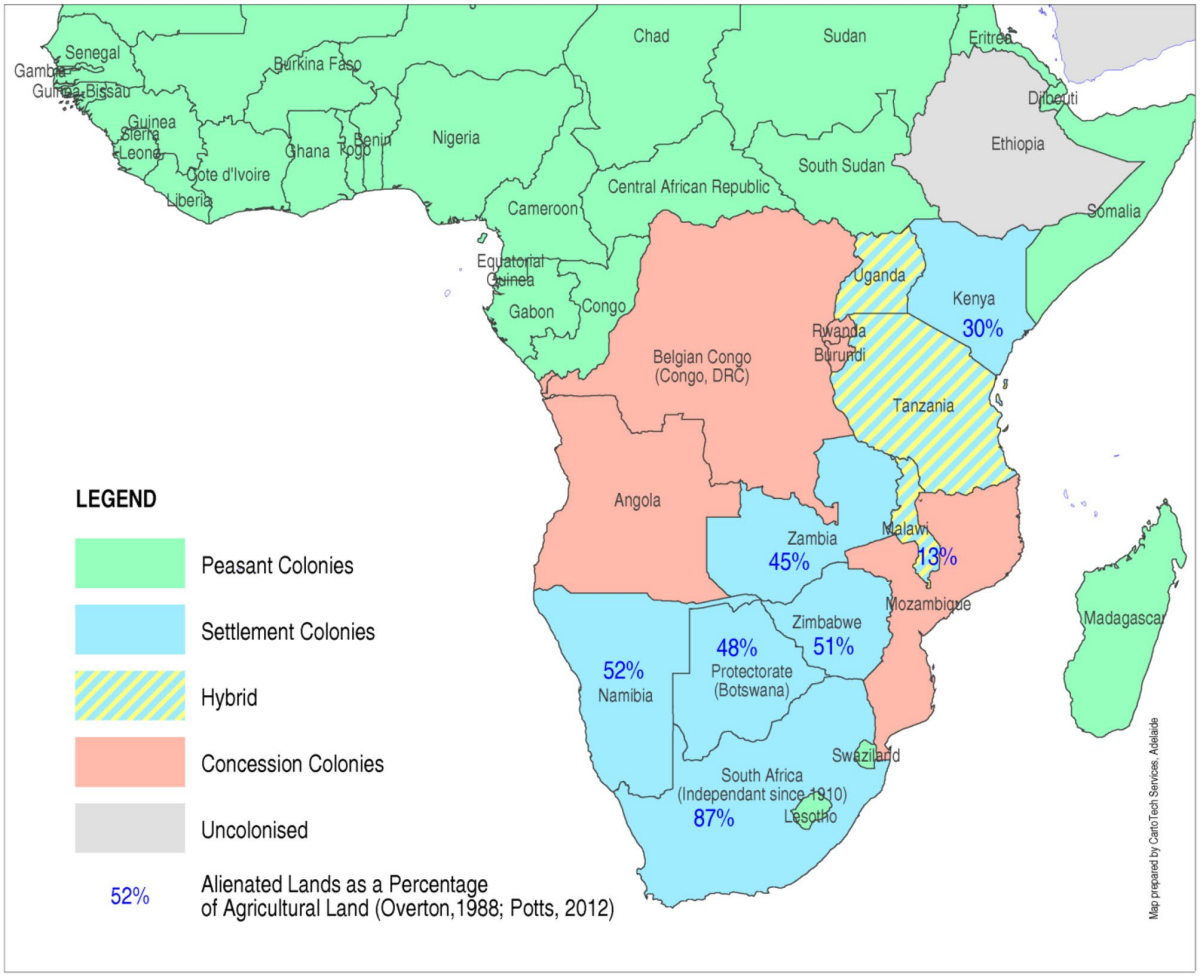
Colonial models ( Source: map compiled by authors)

Railroad construction was expensive in the vast African interior. Hence, colonial investments focused on accessing areas with rich mineral deposits, which could justify the extraction costs (Fig. 1). Export crop production took place where commodities could be cost-effectively transported to railways or harbours, using river transport or bullock teams (Jedwab & Storeygard, 2019). Such regions became the economic zones, generating revenue and discretionary spending power and attracting traders, money lenders, and missionaries who provided schools and health clinics. The hinterland became a source of labour supply and food requisition (Figs. 1 and 2), while the pastoral arid zones provided meat and hides. These areas received no infrastructure investment or services. Depending on existing trade and power relations, three colonial models emerged (Golub, 2015): peasant; settlement (including a hybrid model); and concession colonies (Fig. 3).

Peasant colonies were mainly in Western Africa, where France and Britain continued the centuries-old practice of African merchants buying commodities from peasant farmers or African-owned plantations (Fig. 3). The economic zone on the tropical and savanna coasts, produced cocoa, palm oil and groundnuts, and the hinterland produced groundnuts, long-staple cotton and grain for export or food requisitions (Fig. 1).

The settlement colonies were in southern and eastern Africa where Europeans had extensive investments in land and mines. Settlers secured the most fertile land with reliable rainfall, such as the Kenyan highlands, central Zimbabwe, and the Kafue plains in Zambia (Figs. 2 and 3). Here, settlers gained significant production advantages such as a monopoly on exports, transport infrastructure, property rights, and preferential access to domestic markets through a dual pricing system (Jedwab & Moradi, 2016; Mosley, 1983). Africans in Zimbabwe and Kenya were relocated to Native Reserves, limiting their access to land (Brown, 1968; Deutsch, 2006; Dunlop, 1971). In the hybrid colonies farmers provided a substantial part of the colonial revenue; hence, settlers did not receive the same support and had to compete for markets and labour (Curtis, 2003; Iliffe, 1979). However, this was not on equal terms: for example, Africans in Uganda and Tanzania had *corvée* labour and crop production obligations (de Haas & Papaioannou, 2017; Neal, 1981). In Malawi, settlers struggled to compete with African farmers in tobacco production but secured quotas in export tea-markets (Frankema et al., 2014). The settler areas and transport corridors were the economic zones, allowing African farmers located near railroads access to urban markets.

Concession colonies emerged where powerful Luzo-African merchants continued slave-trading despite the transatlantic ban on slave exports (Pitcher, 1991). Here, Belgium and Portugal granted large land concessions to European companies, with minimum oversight and significant power to raise taxes, delegate land and mining rights and force Africans to work under slave-like conditions (Hochschild, 1998). In Mozambique, forced labour was used in cotton, sugar, or tea plantations (Urdang, 1989). Labour was also supplied to South African mining companies, which provided revenue for the colonial administration. (Alexopoulou & Juif, 2015).

### Impacts of colonialism on food security

**Fig. 4:**
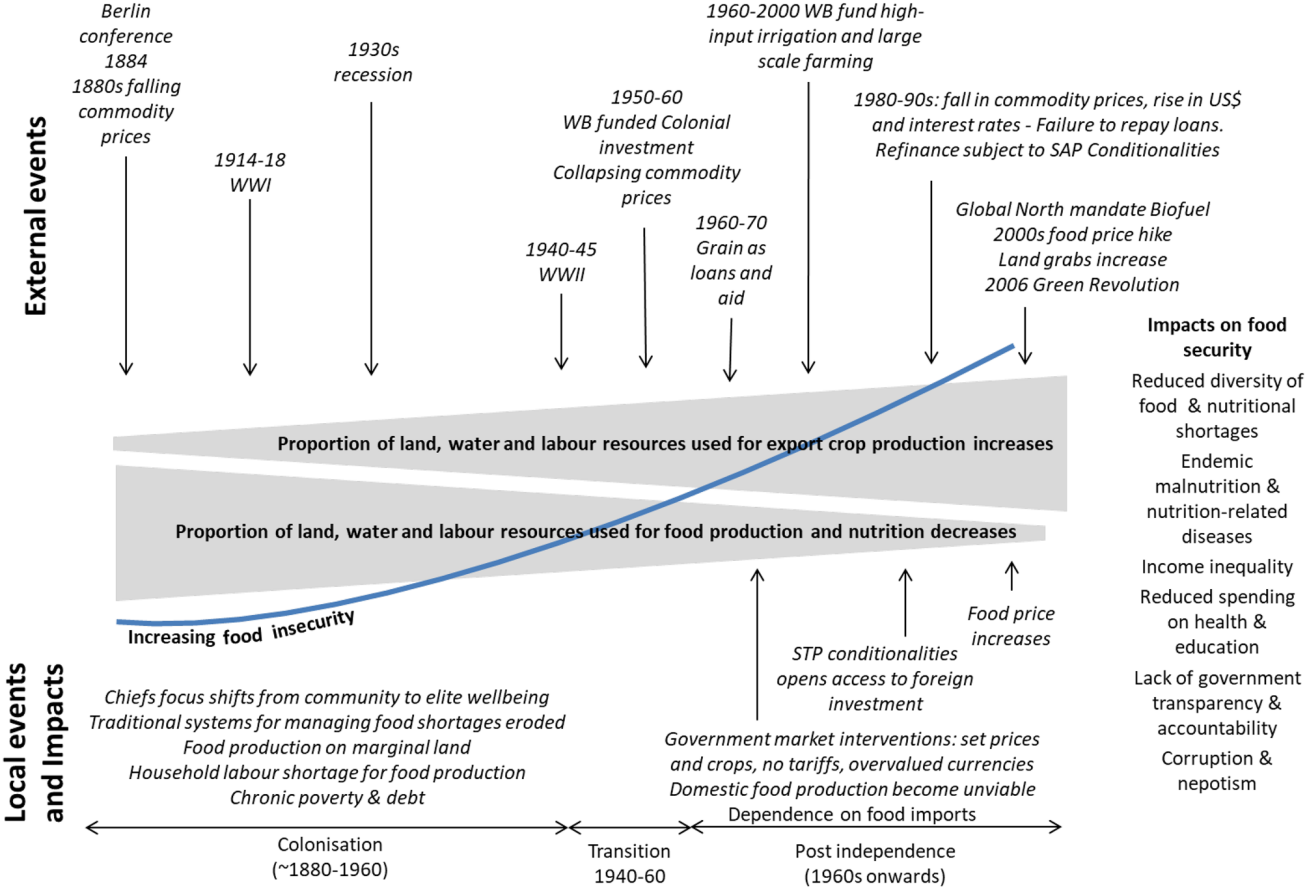
External and local events and impacts that cause increased food insecurity

Growing demand from industries in Europe and North America drove exponential growth in export crops at the expense of food security (Fig. 4). In Western Africa, exports to Britain and France doubled five times and three times, respectively, over a 70-year period (1870 to 1940) (based on Frankema et al., 2018). The area dedicated to export crops in SSA increased by 56% during the 1800s and a further 136% from 1900 to 1960 (Goldewijk et al., 2017). This increase was initially fuelled by high commodity prices, but after 1880 it was driven by the need to off-set the impact of declining prices and the onset of WWI. Hence, the growth in export crop production was driven by colonial demand rather than farmers responding to market opportunities (Frankema et al., 2018). This subsection summarizes six inter-related impacts of this expansion that undermined food security.

#### Reduced food production

As exports expanded, labour was diverted from household food production (Fig. 4). Simultaneously, African farmers were forced to clear more marginal lands for household food production (Hu et al., 2018). Whilst African farmers in the peasant colonies preferred to grow edible export crops for consumption and sale (Jacks, 2012), they were coerced to mainly produce non-edible crops. The export requirement for long-staple cottonto be produced as an annual crop during a short rainy season, rather than a perennial crop— increased the labour demand and reduced manpower availability for food production (Robins, 2016). The impact on cotton growers was compounded by high direct taxes that were not adjusted when harvests failed, and administrations did not provide food assistance (Watts, 1983). As labour availability diminished, deficiencies in food supplies became endemic, distribution systems were dismantled, and important livestock production was marginalized (Darkoh, 1989). Following WWI, urban expansion created dependency on food imports, such as rice, flour, baby milk powder, and tinned fish (Robins, 2018).

Pastoralists in the pastoral region (Fig. 1) were incentivized to supply meat to the economic zone during the 1909–1914 drought. Realising the contribution that this made to the colonial economy, the administration increased the number of wells, provided veterinary services, and opened new markets (Brough & Kimeny, 2004; Swift, 1977). This disrupted the traditional systems of pasture and well regulation. Combined with regulations limiting the mobility of pastoralists, livestock numbers increased and resulted in overgrazing and loss of stock-feed (Sterling, 1974). Conflicts over resource access increased, as did poverty among the owners of smaller herds whose grazing and watering rights were reduced (Alkire & Housseini, 2014).

In the settler colonies of Kenya and Zimbabwe, export expansion caused severe land degradation as farmers and livestock were forced onto reserves, which doubled the population densities between 1911 and 1950 (Mosley, 1983). Poor soils and reduced land availability forced farmers to become labourers, and the sale of produce as a proportion of income halved between 1900 and 1932 (Arrighi, 1966; Glantz, 1976). When guaranteed maize prices were introduced to support settlers during the 1930s, Africans’ crop and cattle sales dropped by 78% and the reserves experienced severe economic regression (Duminy, 2018; Vickery, 1985).

In the concession colonies of central Africa, the system of forced labour and set quotas for rubber collection left no time for food production, and an estimated ten million people died of starvation or ill-treatment in the Congo between 1880 and 1920 (Hochschild, 1998; Vansina, 2010).

#### Loss and replacement of traditional sources of nutrition

Land clearance and mono-cropping destroyed opportunities for grazing, hunting, fishing, and growing nutritious pulses and vegetables. Nutrition and livestock productivity declined (Hu et al., 2018) and income to supplement diets was lost.

Cassava and dent maize replaced more nutritious staples due to their higher calorific content, faster growth, and lower labour requirements (Lappé & Collins, 1982; McCann, 1999). Dent maize was preferred for export as it had higher yields and larger kernels and was more suitable for mechanized milling; however, it was less drought tolerant and more susceptible to pests (McCann, 2007). Serious health issues arose when maize became the primary staple, as it is low in essential amino acids and associated with diarrhoea, dementia, dermatitis, and cognitive deficiencies (Mabey et al., 2014; McCann, 2007; Weis & Büttner, 2018). Malnutrition has long-term impacts on physical development and the ability to pursue education and careers and is therefore an impediment to the prosperity of individuals and households (Alkire & Housseini, 2014).

Food shortages and poor nutrition continued to increase during the 1920s and the calorific intake dropped to starvation level. In response, maize production was further encouraged, which increased the calorie intake but made malnutrition endemic (Nott, 2019; Robins, 2018).

#### Inequality within communities

The wealthy and powerful local elite focused on achieving colonial pursuits rather than community prosperity. In the peasant colonies, the increased demand for export crops, such as cocoa in Ghana, enticed chiefs to ‘sell’ land to wealthy and influential farmers from neighbouring areas, widening the inequality experienced by local farmers and migrant workers (Amanor, 2005, 2010; Amin, 1974; Hills, 1997). The cash-advance crop mortgage system also exacerbated inequality. This system lent money to small-scale farmers unable to fund export production (Austin, 1988; Hopkins, 1973; Watts, 1983). Interest rates varied but were typically 50–100% for cocoa, and up to 200% on the savanna where rain was less certain (Austin, 1988; Thornton, 2014; Watts, 1983). Advances were based on crop estimates and were to be repaid at harvest, regardless of the outcome. Considering the vagaries of late rains, locusts, price variability, disease, and personal calamity, harvests regularly fell short of estimates. Farmers had to borrow more money at escalating rates, creating a vicious circle of borrowing and debt re-payments (Haswell, 1975).

Merchant manipulation of scales, transport prices and chiefs’ timing of tax payments are further examples of how the elites widened inequality and created dependency. For example, farmers in the Sahelian savanna sometimes received only 26% of export prices, and Nigerian farmers had to sell their stored grain to pay taxes that chiefs demanded before the cotton harvest (Watts, 1983). Impoverished households disintegrated into individual labourers, while better-resourced farmers and pastoralists prospered (Dahl & Hjort, 1979; Shenton & Lennihan, 1981; Shipton, 1985).

In the settler colonies, the favourable conditions granted to European famers were accompanied by policies that disadvantaged African farmers. While African maize farmers on the Kafue floodplains successfully competed with settlers to supply mines, the administration alienated their land when demand for maize reduced during the 1930s. This forced farmers to work on settler farms and public infrastructure projects (Makings, 1966; Vickery, 1985).

The colonial production and trading system monetized social relations and outcomes, and communities lost the capacity to control their productive resources and farm sustainably (Arnold, 1988). The cumulative effect was a concentration of wealth, land, livestock and trees with large-scale farmers and the local elite. The complicity of the chiefs ensured the profitability of merchants, which increased inequality within communities (Amanor, 2007; Austin, 1988). As a result, the pre-colonial food security mechanisms were destroyed; hence, when harvests failed or calamities occurred, the effect was no longer mitigated within the community.

#### Spatial inequality

Across SSA, spatial inequality arose through economic and infrastructure disparities between the hinterland and well-serviced economic zones and urban areas (Johnson, 1933).

In the tropical forest of the Gulf of Guinea, the high-value export crops (e.g. Cocoa) raised most of the administration’s revenue and farmers paid little direct taxes. Whereas cotton and groundnuts raised less revenue and savanna farmers paid higher direct taxes. When the commodity prices plummeted in the 1930s, cotton growers paid 30% of their profit in taxes and many males migrated to work in the cocoa areas (Ochonu, 2006). Households relied on migrants returning with cash and labour, and experienced food shortages if their return did not coincide with the opening rains.

The northern pastoral zone of the Sahel remained the least developed and most impoverished, and therefore most affected by malnutrition and disease (Hunter, 1967). The hinterland also received the least education as NGOs were less active in remote regions (Mosley, 1983; Punt, 1979). In 1940, English literacy in Nigeria was 20% in the south and 2% in the north (Kohli, 2004). This created spatial inequality as proficiency in the colonial language was essential to operate in the colonial system (Yates, 1996).

In the settlement colonies of Kenya and Zimbabwe, inequality emerged between the settler areas and rail corridors, and the native reserves and hinterlands (Fig. 2). Reserves were mainly on land unfit for agriculture, with insufficient rainfall (Glantz, 1976), small plots, minimal services, and restricted freedom of movement.

#### Diminished productivity and food reserves

Food reserves diminished in a vicious cycle of income loss, declining productivity, and increasing debt. High protein crops (sesame, peanuts, and pulses) were sold to pay taxes and debts (Little, 1991). Further, mono-cropping reduced fallowing and, in combination with farming of marginal land, depleted soil fertility and yields. Hence, farmers could not afford to replace the lost soil nutrients (Hu et al., 2018). Lower productivity and the inability to pay debts further increased the pressure to sell stored grain, and limited investment in inputs (Darkoh, 1989; Ker, 1995; Wade, 1974).

In peasant colonies, grain reserves were undermined by intensified food requisitioning, which had priority over household’s food security (Watts, 1983). For example, the expansion of tin mines on the Joss Plateau (Nigeria) in the early 1900s, tripled the food requisitions demanded from the supplying communities (Stilwell, 2011). In settlement colonies, the dual market system decreased African farmers’ trade and undermined their ability to invest in improved productivity (Makings, 1966; Overton, 1988). Labour demand for the mines and the need to earn cash resulted in an absence of males in 60–70% of African farm households, which further reduced their productive capacity (Palmer, 1983).

Productivity and grain reserves were further diminished during periods of war and recession in Europe: adult males were conscripted, food requisitioning and exports intensified, and food prices and direct taxes increased (Killingray, 1987; Mackenzie, 1998). As food shortages worsened, diseases–the Spanish Flu, tuberculosis, and malaria–spread through populations already weakened by famine (Maxon, 2000), further reducing the productive capacity of farmers.

#### Regression of the domestic economy

Pre-colonial African societies were able to diversify and safeguard their domestic needs during their early maritime trade with Europe. However, the colonial system decimated domestic food production systems, supply chains and markets (Jedwab & Moradi, 2016; Worboys, 1988). Africans’ direct participation in global markets was severed, and they could no longer respond to volatile commodity prices by developing new markets and alliances. In the absence of rural economic development, local labour markets and manufacturing declined, and the domestic economy regressed.

## Transition of power: international influence and lack of accountability

SSA’s transition to independence mainly took place from 1955 to 1965. It was dominated by the metropoles’ desire to protect their investments in land, minerals, and export crop production (Keane et al., 2010). The first sub-section discusses how the metropoles assured their position in the ‘dying days of colonialism’, and the second covers the role of international institutions and the World Bank’s (WB) growth strategy.

### The dying days of colonialism

Opposition to colonialism, in the colonies and Europe, increased in response to persistent reports of poverty and lack of access to basic needs, such as nutritional food, health, and education. Hence, public spending on social services and food imports quadrupled per capita in British and French colonies between 1945 and 1955 (Hodge, 2007). As it became clear that colonialism was untenable, the metropoles worked to secure and increase extraction of export crops post-independence (Singh & Ovadia, 2018, Fig. 4):

In the peasant and settlement colonies during the 1950s, the metropoles embedded the African elite in western approaches and values. They were assimilated into the colonial administration and trained in its systems. Therefore, the new nations largely retained the colonial administration systems (van de Walle, 2009). Sons of the elite were sponsored to attend universities in the Global North, which made them supportive of its technologies, strengthened their mutual economic and cultural interests, and distanced them from developing African solutions to African problems (Samoff & Carrol, 2004).

By 1955, Britain had planned or implemented 55 projects to increase export crop production, including infrastructure, irrigation, and mechanization (Heldring & Robinson, 2012). Projects were designed using western experts and technology, but with no consultation to understand local conditions. Many projects failed, leaving post-independence nations with the debt and non-productive assets (Hodge, 2007; Li, 2005; Shanguhyia & Falola, 2018). Unfortunately, these lessons were not learnt, and a development paradigm was established that continued post-independence (Higginbottom et al., 2021).

In 1961, France took control of the national currency reserves of fourteen West African countries, retaining considerable control over their spending (Taylor, 2019). The metropoles and international firms secured further concessions and registered businesses locally. In Tanzania and Kenya (1940–1960), Europeans companies gained new timber concessions and created National Parks, which forced communities and their cattle from forest and savanna areas (Conte, 2004).

### International development, financial governance, and trade policies

After WWII, the US became the global leader, and the US dollar became the currency of international trade (Campbell, 2017). The US government decided to use the chronic overproduction in the US food system to pursue global strategic goals (Campbell, 2017; Davis, 1986): securing US access to resources; disposing of US grain as food aid; and restricting the Soviet Union’s access to resources (Makki, 2015). The US intentions were clearly expressed by the Vice President of the WB in 1972: “the main objective for foreign assistance is to produce the kind of political and economic environment in which the US can best pursue its own social goals” (Toussant, 2008, p. 100). Three international institutions were created: the WB and the International Monetary Fund (IMF), as lenders and monitors of financial and economic policies (Hodd, 1987); and the General Agreement on Tariffs and Trade to minimize barriers to international trade (Cheng, 2007).

The WB and IMF food programmes and trade policies made self-sufficiency in food production unviable (Mercier, 2020), which destroyed livelihoods and created import dependency (Ahluwalia, 2019). International trade regimes did not incorporate fair-trade agreements for the Global South (Sharma, 1994). Hence, discriminatory practices deprived African exporters of millions of dollars, resulting in financial dependency (Gunewardene, 1991).

The WB provided funding subject to its political and economic growth agenda: funding infrastructure to encourage private sector investment in production, marketing, and distribution of industrial and agricultural products (Kapur et al., 1997). The WB strategy had four steps: 1) increase agricultural production and productivity using new technologies, such as perennial irrigation and mechanisation of large farms; 2) generate export earnings, jobs, tax revenue, and a positive trade balance; 3) integrate the domestic agricultural sector into the national economy to support local manufacturing and create jobs throughout the economy; and 4) provide raw materials for industrial production, domestic consumption, and export (Timmer, 1988; Zoellick, 2010).

The Global North made a conscious decision that the new nations should pay the cost of addressing the colonial underdevelopment, repay the loans for the failed developments of the 1950s, continue to accommodate the needs of the Global North (Adams, 1992), and pay compensation to re-possess alienated land (Hazelwood, 1985). This curtailed the new nations ability to invest in rural economic development.

## Post-independence: geopolitics and the persistence of food insecurity

SSA governments assumed leadership of impoverished economies with little public finance, domestic savings, or infrastructure (World Bank, 1994). Independence did not achieve the cultural, political, and economic changes that were necessary to ensure that governments linked economic development to societal objectives and met citizen’s needs (Englebert, 2009; Maathai, 2007).

Africa is the only region in the world where increased export production (1980s and 90 s) caused a decline in per capita food production (Dietz, 2013). Even though most African countries were net exporters of agricultural products, cereal imports grew from 2.5 to more than 15 million tons between 1960 and 2000, and by 2010 SSA’s average per capita income was less than half that of other developing countries (Chauvin et al., 2012). Poverty remains the main barrier to access food. In rural areas in particular, millions are at the mercy of urban-biased distribution, foreign aid, commodity speculators, foreign exchange fluctuations and the geopolitics of global food trade (Dodo, 2020; Ringler et al., 2010). Colonialism had laid the foundation for globalization of food systems (Friedmann & McMichael, 1989).

As agricultural production was the only sector with institutional support and infrastructure, Step 1 of the WB’s strategy resulted in export growth, but reduced food production and created a dependency on imports and aid (discussed in Sects. 4.1 to 4.4). Consequently, the anticipated benefits of Step 2 did not materialize. Section 4.5 discusses the failure to integrate the agricultural sector into the national economy and to provide raw material for manufacturing (Steps 3 and 4). Section 4.6 discuss the foregone opportunities to create food security.

### The export focus led to dependency on food imports and food insecurity

The continued dependence on export production to fund development and service loans (Cashin & Pattillo, 2000; Lofchie, 1975) proved to be an unviable strategy for the following reasons (Fig. 4):

- Market price volatility: when commodity prices collapsed in the late 1950s and 1960s (World Bank, 1983) farmers were encouraged to expand export crop production; for example, between 1958–1966 Ghana’s cocoa production doubled and occupied 50% + of arable land; similar increases for tea and coffee in Kenya (Lappé & Collins, 1982); and by 1976 groundnuts in Gambia and Senegal occupied 70% and 55%, respectively (Collion, 1981; Darkoh & Ould-Mey, 1992).
- Governments’ acceptance of cereals for almost half of US loans and grants: these were sold at or below African production costs, putting downward pressure on local prices and making local production unviable (Adams, 1992; Boussard et al., 2006; Morgan, 1963; Singer, 1989).
- Governments’ intervention in markets to keep prices of staples low and improve access to food: by maintaining overvalued currencies and zero tariffs on food imports and prescribing crops and setting prices, created a disincentive for investment in domestic food production and markets and encouraged export crop production (Bates, 1981; Goyal & Nash, 2017).
- Floating exchange rates: in the late 1970s these caused increased living costs as US food imports exceeded the value of Africa’s agricultural exports (Faostat, 2011; Lofchie & Commins, 1982).

These factors combined to decrease domestic food production. For example, food production in Mali had declined to a quarter of 1967 levels by 1975. This was exacerbated by soil degradation from the use of increasingly marginal land, and post-harvest losses due to insufficient storage capacity (Cohn, 1975; Herrmann et al., 2005; Suleiman et al., 2015).

### Large farm policy

Governments used international loans to subsidize land, fertilizer, credit, and other inputs for large farms that were mainly on newly cleared land. This land was granted to the elites who received a large share of governments’ agricultural development funds; for example, 50% and 90% in Nigeria and Ghana, respectively (Bates, 1981; Roider, 1971; Williams, 1976). Similarly, the mechanized grain farmers in Northern Ghana received cheap land for rice production from the 1960s. Despite their land only being 20% of arable land in 1974, they received 75% of imported fertilizers, all the improved seeds, tractors at subsidized prices, and 56% of available credit (Kline et al., 1969; Rothchild, 1979; USAID, 1975 cited in Bates, 1981). These subsidies secured elite support for governments but did little to improve food production or support rural communities (Boussard et al., 2006). Mechanization was also economically unviable when commodity prices were low (Bates, 1981). The large farm policy was costly, biased against the small farm sector, and failed to reduce food imports (Djurfeldt et al., 2005).

### Large irrigation schemes

Large irrigation schemes with dams and hydropower are complex and costly ways of increasing production. Their success depends on the ability of: i) governments to manage the complexity and repay the investments; ii) farmers to access and pay for the necessary inputs and transport their produce to market; and iii) markets to charge prices that reflect these costs. The socioeconomic conditions in SSA in the 1960s–very low-income levels per capita, high fertilizer prices, lack of infrastructure, and government market interventions–meant irrigation was not viable (Bjornlund et al., 2020b). Fertilizer prices in Africa are at least double those in Asia, and the average application rate has remained low as it is not economically viable: for example, 14 kg/ha compared to 141 kg/ha in South Asia (Bumb & Baanante, 1996; World Bank, 2018).

Irrigation schemes on the Sahelian floodplains failed to increase production due to: remote locations, inadequate infrastructure and market access, top-down management, poor design, lack of data on watershed hydrology and technical issues (Bjornlund et al., 2020b; Higginbottom et al., 2021). Additionally, dam construction flooded upstream farmers’ land, and reduced the area available for flood-recession dowwnstream. Hence, more farmers lost livelihoods than gained access to land on the schemes (Adams, 1992). FAO (2016) estimates that only 54% of the land in the Sahel schemes was used.

A comparison of irrigated mono-rice production with the diverse production of traditional agricultural water management, found rice production had minimal economic benefit (3.9%), presented considerable financial risk, and created a nutritional deficit (Drijv-r and Marchand (1986). The anticipated double rice cropping was not practiced as it conflicted with the higher income from livestock rearing (Adams & Grove, 1984).

With over half the audited large-scale irrigation schemes failing between 1965 to 1986, the WB started to support small-scale schemes (World Bank, 1988; Toussaint, 2007). However, they faced similar issues and fell into disrepair (Bjornlund et al., 2017). Rather than increasing production, irrigation schemes created government debt and food insecurity for hundreds of thousands of displaced households (Adams, 1993).

### The World Bank, IMF and World Trade Organization (WTO)

The debt crisis in SSA was driven by macroeconomic developments in the Global North. Between the early 1970s and 1982, the economic future of the Global South was seriously affected due to the recession that followed the oil crisis, which resulted in escalating current account deficits, and worsening terms-of-trade. Critics of the WB argue that the economic dependency and failures of African countries could have been avoided if it had reformed its lending practices, funded projects that strengthened the domestic economies, and created fair trade agreements for Africa’s exports (Adams, 1992; Sarris & Sharns, 1991).

The WB and commercial lenders funded projects without sound evidence of their viability or accountability for expenditure (Adams, 1992; Jones, 1995). Many countries in SSA failed to service their loans due to imprudent spending, floating exchange rates, appreciation of the US dollar (1980–85), high interest rates (Little and Watts, 1994), and declining commodity prices from 1980. From 1980 to 1998, SSA repaid four times its original debt, yet the debt tripled (Ismi, 2004; Iyoha, 1999).

Refinancing was subject to the conditionalities of the IMF’s Structural Adjustment Policies (SAPs); for example, allowing foreign companies access to natural resource, and drastically reducing spending on food imports, agricultural subsidies, education, health, and housing (von Braun & Meinzen-Dick, 2009). Foreign companies further expanded export crop production (Daniels et al., 2016; FAO, 1996) by clearing land or converting agricultural land to production of biofuel, animal feed and carbon off-sets. This contributed to the global commodity crisis of 2006–8 (Beyene, et al., 2013; Jindal et al., 2008; Wily, 2013), which triggered a surge in demand for agricultural land in SSA (Rullia et al., 2013), food shortages, rising food prices and loss of livelihoods (Tchoungui et al., 2020). Land acquisition in Africa has created little local economic development (Tchoungui et al., 2020), and estimates range from 15–20 million ha (von Braun & Meinzen-Dick, 2009), to 56 million ha (Deininger et al., 2011) and up to 230 million ha (Krugelman & Levenstein, 2013). The SAP-enforced spending cuts have had negative long-term impacts on infrastructure investment, transport prices, market integration (Riverson et al., 1991), education, and health. These cuts coincided with the AIDs epidemic, which infected an estimated 15 million people in Africa (Gordon et al., 2003), and reduced the labour force in many rural areas.

The WTO policies allowed governments to subsidize their agricultural producers. This reduced global commodity prices and undermined ‘comparative advantages’ for African producers. For example, Burkina Faso and Chad lost US$ 13.7 million in cotton export earnings in 2002 due to US cotton subsidies (Oxfam, 2004), which equalled 21% and 33% respectively of their debt payments. While developed countries could afford the subsidies, the SAPs denied SSA countries this opportunity (Wise, 2004; Woodward, 2007), furthering market inequality.

### Failure to integrate the agricultural sector into the national economy

SSA governments failed to strategically invest in rural economic development and to facilitate the integration of food production into regional economies. This made it difficult for state agencies to secure cereal supplies (Watts, 1983), and for industries to secure access to local produce of sufficient quality and consistency. These issues contributed to foreign investment being biased towards imported inputs and technologies, creating barriers to integrating agriculture into the economy and generating economic growth and jobs (Bates, 1981; Mendes et al., 2014). Manufacturing accounted for less than one percent of the workforce in 1976 (Fransman, 1982). Despite the sector growing by 7% per annum between 1960 and 1980, SAP-driven privatization caused a decline in manufacturing and detrimentally impacted consumer spending on food (Mendes et al, 2014).

In the absence of government initiatives, small-scale food production, trading, regional distribution, and transport providers emerged (North, 1990) within an informal economy. Despite volatile and weak legal systems, this economy grew incrementally and has generated an estimated 50–75% of employment and supplied food to urban and rural households (African Union, 2008; Hann, 2006).

### Foregone opportunities to create food security

At the time of independence, there were about 33 million small-scale farms in Africa (IFAD, 2013), individually cultivating less than 5 ha but accounting for over 80% of farms and approximately 90% of agricultural production (Asenso-Okyere & Jemaneh, 2012; Wiggins & Keats, 2013). Despite this, small-scale farming was not included in the WB strategy or governments’ plans for development and food security (Lappé & Collins, 1982). They remained under-resourced, and food programmes and government commodity boards undermined their viability (Singer, 1989).

The following two sub-sections summarize some of the most critical opportunities that were foregone to increase food production. Had they been facilitated the small-scale farming sector could have made a significant contribution to food security and the national economy. While the focus is on small-scale farming, much of the discussion also applies to medium and larger farms.

#### Information and infrastructure

Lack of government-supported information channels and institutions created barriers to farmers’ productivity. It hampered innovation, private investment in facilities and vocational training (UN, 2015; Quarteya, et al., 2017). Farmers’ productivity could have been improved if government policies had facilitated the development of:

- systems providing information flow between farmers and input-suppliers, output-buyers, and transport providers about availability, demand and prices in different markets (Maur, 2008; Vorley, 2013);
- storage and packaging facilities to reduce post-harvest losses and roads for transportation of produce to markets, which allows farmers to sell produce of acceptable quality and when prices are higher (Burke et al., 2019);
- holistic training of extension officers to share experiences between farming communities and researchers, and provide advice on aspects such as soil management under different cropping rotations, improved pastures, inter-cropping, market access, and profitable crop selection (Msuya et al., 2017); and
- improved market access to ensure increased profitability and return on labour (Abdoulaye et al., 2014; Agbamu et al., 1996).

#### Research and development into locally appropriate production systems

Funding for agricultural research in Africa during the colonial period was biased towards improving export crops, with work on food crops limited to crops that required less land and labour (Bjornlund et al., 2020a, b). The first international agricultural research institutes to address concerns about population increases and food security in the developing world were established in the 1960s. The International Rice Research Institute (IRRI) in 1960 and the International Maize and Wheat Improvement Center (CIMMYT) in 1963. These were later incorporated into the Consultative Group on International Agricultural Research (CGIAR), which had 13 research institutions by 1983. Initially, the approach was primarily biological and technological (Richards, 1985). In more recent decades, more attention has been given to farming systems for specific agricultural zones and economic and social factors. However, the lack of markets and information flow (Sect. 4.6.1) were barriers to the effective incorporation of research into farmers’ fields, and the new varieties sometimes did not produce well under field conditions.

The focus on export crop production meant that economic, social and political resources were unavailable to address the research and development needed to develop national food security, such as:

- production systems, suitable for the diverse biophysical and socioeconomic conditions across SSA, to increase productivity on the heavily leached and nutrient-poor soils, which lacked organic matter to make chemical fertilizer available to the plants;
- varieties of small grains and pulses to improve the nutritional value of diets and provide the regionally preferred food;
- crop-livestock integration to improve household income and soil fertility;
- efficient and cost-effective farm implements and other technologies to increase farm productivity and income, which are suitable for small plots and that communities can afford, understand and repair (Dumont, 1966; Pingali, 2012); and
- small scale, community-based value adding processing to increase farmers’ income and generate jobs within rural communities.

#### Geopolitical institutions

When the United Nations and the WB were formed, two critical institutions were proposed to address the economic disparity created by colonialism, but they never eventuated due to US and British opposition: the World Food Board, to provide global supply management to avoid price fluctuations caused by scarcity and gluts of essential foods; and the International Trade Organization. Such institutions could have balanced the tension between national regulation in pursuit of food sovereignty and trade policies to dispose of food surpluses under liberal trade policies (Friedmann, 1990).

## Discussion and conclusions

The colonial system was export-oriented often with inappropriate production systems, which were politically motivated rather than promoting rural economic development and food security. This resulted in severe local food shortages and a movement away from traditional mixed production systems. Post-independence, the newly established governments considered Western agricultural production systems and technologies as the best way to achieve food sovereignty. This included production of wheat, maize and rice, which were not the preferred staples in SSA. In doing so, governments failed to achieve food security for households and communities. This high-input high-output production model proved incompatible with Africa’s biophysical and socioeconomic conditions. The associated risk, labour demand and mechanization needs were unsuitable, particularly for small-scale farming. Without the enabling state-driven market institutions that accompanied the Green Revolution in Asia, these production systems have not led to increased productivity and growth in the domestic agricultural sector in SSA (Djurfeldt et al., 2005).

Food shortages did occur in pre-colonial times and environmental stochasticity impacted production and livelihoods. However, production, trading and social systems had evolved along trajectories driven by local context. Hence, food shortages were transitory rather than permanent. The imposition of external demands and western agricultural production approaches drastically disrupted these systems through three key impacts. First, food production declined due to the allocation of scarce fertile land and labour to export crops, without improving land and labour productivity. Second, the focus of traditional leaders shifted from community well-being to the wellbeing of the elite. This undermined the social contract between leaders and communities. Hence, the local elite resisted the need for fundamental economic change post-independence. Third, US-imposed trade policy tools, which reduced livelihood opportunities, displaced ecologically sustainable agricultural practices, and created import dependency (Koning, 2017).

Inappropriate lending by the WB left SSA nations indebted and without productive assets to service their debts. In response, Structural Adjustment Policies (SAPs) forced nations to cut spending on agriculture, education, housing, and health, and allowed private foreign investment to extract the resources that underpinned local communities’ livelihoods. A vicious circle of debt and interest payments drained SSA of cash for domestic development. This kept Africa in poverty and remains a barrier to the development of cost-effective and locally appropriate domestic food production and supply chains.

The SAPs forced governments to allow commercial land acquisitions by international companies and local investors, which further undermined food security and livelihoods. Land transfers to these businesses often failed to acknowledge local land-users (Jayne et al., 2019). Consequently, food producing farmers were evicted from their lands (Tenenbaum, 2008) and the land used for non-food crops.

The increasing price of imported food since 2000 has encouraged local farmers and developers to invest in farming. Jayne et al. (2019) finds that the number of farms of 5 to 25 ha has increased. This has been driven by new players with money often earned from non-farm activities. This presents an opportunity for improving productivity and viability. However, there is also the potential for displacement and loss of livelihoods for local communities through deforestation, environmental impacts, and transfer of farmland from domestic food production to export crop production (Huggins & Clifton, 2011). More recently, the region has become vulnerable to international and domestic supply chain disruption and delays, and restrictions on movement and export of food in response to COVID-19 (George, 2020; Hamilton, 2020).

Achieving food security requires farmers and rural communities to be integrated into income generating activities so they can purchase the food and services they need. Not all small-scale farmers can become viable, and land consolidation is needed for farmers with productive capacity to expand and specialize. This process is politically sensitive in SSA, where private land title was uncommon until recently. Even if possible, many unviable farmers will be reluctant to sell as there are no alternative livelihood options for their household; staying on the farm is the default option. Forcing the process would generate a wave of rural to urban migration, escalating urban slums and poverty. Land consolidation needs to take place in a socially viable way and parallel to the creation of alternative livelihoods.

Farming needs to be profitable for farmers, not just productive. The focus on increasing yields using expensive inputs is not economically viable (Bonilla-Cedrez et al., 2021; Sasson, 2012). Reflecting the foregone opportunities (Sect. 4.6), more inclusive and cost-effective approaches are needed (van Rooyen et al., 2021): for example, i) regional public or private grain storage, reducing post-harvest losses and allowing farmers to keep their harvest until prices increases; ii) improved understanding of intercropping and crop-livestock integration; iii) improved financial, food literacy and education, shifting crop choices from low to high-value and nutritious crops; iv) local value adding processes and linkages to local and urban markets; and v) local phone apps linking farmers with veterinarians, markets, transport providers, and agronomists. A critical component of an agricultural production system is a well-trained and functioning extension system that helps farmers make better enterprise selections based on market signals. This requires training in the business aspects of farming as well as agronomics.

Africa does not have to follow the route of western industrialized agriculture. There has been an increasing trend to focus on crop-livestock integration by developing the discourse of agroecology and reorganizing food systems towards circularity (Van Zanten et al., 2019). However, it is still uncertain whether this will find political traction. There has also been a push by philanthropists and the WB to broker investments in improved hybrid and GM seed technology and linking farmers to value-chains to improve productivity. However, this could embed many farmers in dependency on agro-inputs and expand food exports at the expense of local food security (Daño, 2007; McMichael, 2013). The continent can take note of these initiatives and realign their agricultural and rural development policies closer to a modern version of traditional African systems. These would be more sustainable and economically feasible and would better serve the food security and social needs of the Global South. Future research and development should focus on food production models that are inclusive of small- and medium-scale farmers and integrate components of the system into economic development (Bjornlund et al., 2021). However, this requires African governments to extract themselves from the debt traps and the SAP’s that restrain governments’ ability to support agriculture. African governments could then invest the current loan repayments into their own countries and develop their people and resources along their own development trajectories.

## Data Availability

Not applicable.
